# Retrospective evaluation of pain in patients with coccydynia who underwent impar ganglion block

**DOI:** 10.1186/s12871-020-01034-6

**Published:** 2020-05-11

**Authors:** Ozlem Sagir, Hafize Fisun Demir, Fatih Ugun, Bulent Atik

**Affiliations:** Department of Anesthesiology, Balıkesir University Health Application and Research Hospital, 10100 Balikesir, Turkey

**Keywords:** Coccyx, Chronic pain, Sympathetic nerve block, Pain management, Pulsed radiofrequency treatment

## Abstract

**Background:**

We aimed to evaluate pain scores one year after impar ganglion block in patients with coccydynia who did not benefit from conservative treatment.

**Methods:**

The medical records of 29 patients with coccydynia were reviewed. Patients who were referred to the algology clinic and underwent impar ganglion blocks were retrospectively evaluated. Demographic data, time to the onset of pain, causes of pain, X-ray findings, administered invasive procedures, and visual analog scale (pain) scores were recorded.

**Results:**

A total of 29 patients were included in the study, 10 males (34%) and 19 females (66%). The average age and body mass index were 53.45 ± 9.6 and 29.55 ± 4.21 respectively. In 21 patients, the onset of pain was associated with trauma. Nineteen patients (65.5%) had anterior coccygeal angulation. The average visual analog scale score before undergoing an impar ganglion block was 7.4 ± 1. After the procedure, the scores at < 3 months, 3–6 months and 6 months-1 year follow-up intervals were significantly lower (*p* < 0.05). Furthermore, visual analog scale scores at the 3–6 months and 6 months-1 year periods were significantly lower in patients who received diagnostic blocks plus pulse radiofrequency thermocoagulation than in patients who underwent a diagnostic block only.

**Conclusions:**

The impar ganglion block provides effective analgesia without complications in patients with coccydynia. Pulse radiofrequency thermocoagulation combined with a diagnostic block prolongs the analgesic effect of the procedure.

## Background

Coccydynia is defined as pain in the sacrococcygeal region. It is a disorder that not only reduces a patient’s quality of life, but it is difficult to treat [1]. Numerous physiological and psychological causes can contribute to its etiology. Typically, it is related to chronic inflammation triggered by abnormal mobilization of the coccygeal structures. Childbirth as well as minor but repetitive and direct trauma are considered as possible causal factors. Coccydynia is rarely related to neoplasms or psychological conditions such as somatization [2, 3]. Its incidence is unknown, but female gender and obesity increase the risk [4].

Patients with coccydynia typically complain of pain in the coccyx. This pain increases with prolonged sitting, leaning backwards during sitting, prolonged standing and standing up after being in a sitting position. Conservative treatments such as non-steroidal, anti-inflammatory drugs (NSAIDs), levator ani relaxation exercises, sitting cushions and transcutaneous electrical simulation have all been used to alleviate the pain, however these methods are ineffective in 10% of patients [2].

The impar ganglion, also known as the Walther ganglion, is the terminal pelvic division of the sympathetic chain and is located behind the rectum, anterior to the sacrococcygeal joint and coccyx. It provides nociceptive and sympathetic innervation to the perineum [5]. When conservative treatments fail to alleviate pain in patients with coccydynia, the impar ganglion can be blocked through the sacrococcygeal or the intercoccygeal junction with the help of screening techniques. After insertion and confirmation of the needle position, local anesthetic drugs, either with or without steroids are injected and thermal ablation via radiofrequency may be applied. However there is limited clinical evidence to support the efficacy of these interventions [6].

The aim of our study was to evaluate pain scores in patients with coccydynia who underwent an impar ganglion block in our algology department due to the failure of conservative treatment over the course of a 1-year follow-up period.

## Methods

The medical records of 29 coccydynia patients treated with impar ganglion block at the algology department of Balikesir University Hospital were retrospectively reviewed. Age, gender, height, body weight, the onset of pain and its possible causes (falls, accidents, cancer, or idiopathic) were collected from medical records. X-ray images of the coccyx were examined to evaluate coccygeal structure and underlying physiology and potential pathologies. The images were classified as either normal or exhibiting anterior coccygeal angulation. Our study was approved by the institutional ethics committee (decision No: 2018/156).

The number of impar ganglion blocks used on patients, the 0.25% bupivacaine+ 40 mg methylprednisolone mixture used, and the application of radiofrequency thermocoagulation (RFT) were recorded. Visual analog scale (VAS) pain scores were acquired from the records both before and after the procedures. The VAS scores were obtained during policlinic check-ups and phone calls at < 3 months, 3–6 months and 6 months-1 year time intervals following the procedure. Patients who were referred to surgery after the procedure were also recorded, along with their VAS scores.

**Fig. 1:**
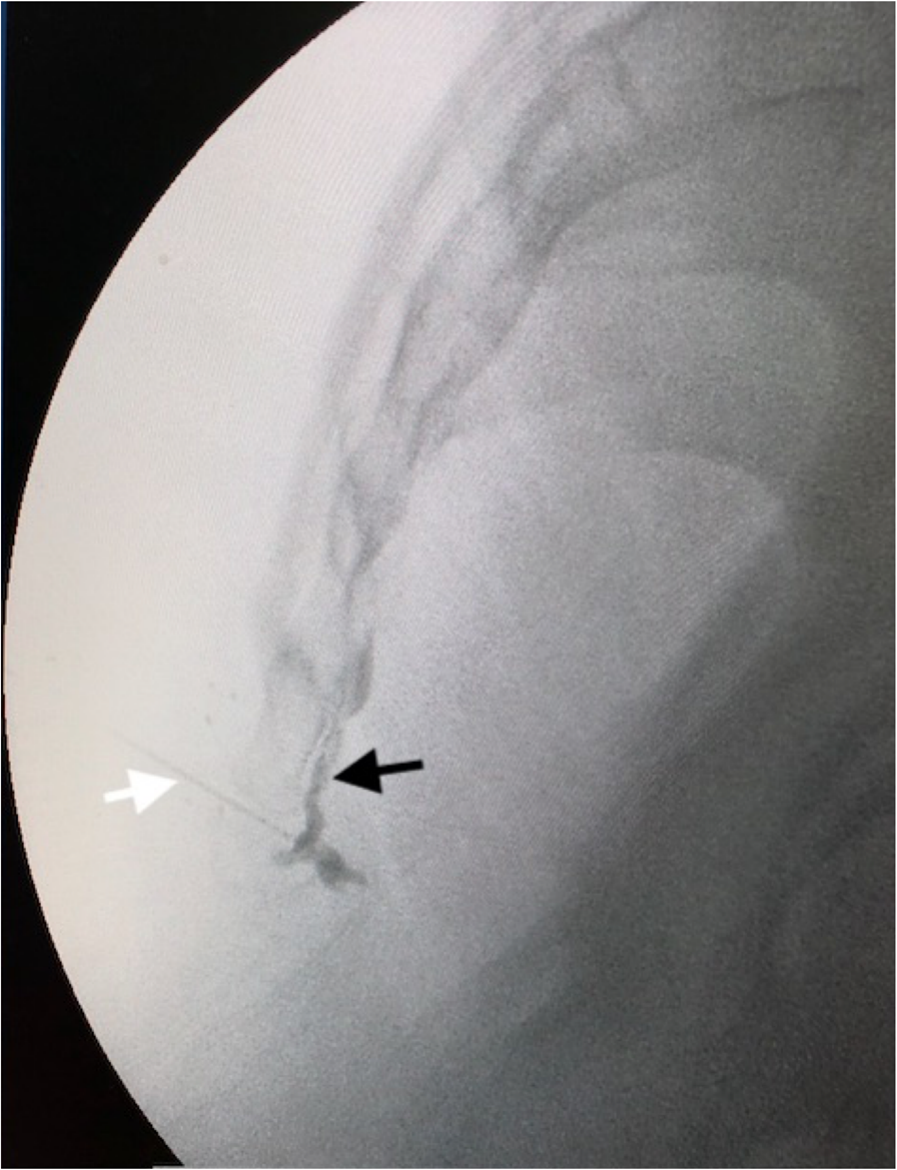
Lateral fluoroscopy view of the impar ganglion block. Application of the needle from the sacrococcygeal junction (white arrow) and confirmation of the location of the needle tip and spreading of the radiopaque dye in the anterior part of the sacrococcygeal joint (black arrow)

The impar ganglion block was performed using a 22-gauge needle from the midline. The tip of the needle and the spread of the radiopaque substance in front of the coccyx were observed using fluoroscopic lateral images with C-arm fluoroscopy assistance (Fig. 1). Blocks performed total 10 mL volume with 0.25% bupivacaine+ 40 mg methylprednisolone were classified as a diagnostic impar block (DB). After the DBs were performed, patients who subsequently underwent pulse RFT (NeuroTherm NT 1100, USA) at 42 °C for 2 min with a 22-G insulated RFT needle with a 5 mm active tip were classified as DB + RFT.

Data was analyzed using IBM SPSS Statistics version 22 (IBM Corp., Armonk, NY, USA). Continuous data is expressed as averages and standard deviations and categorical data as frequencies and percentages. The Shapiro-Wilk test was used to evaluate the normal distribution of parameters. Comparisons of the data was performed using the Wilcoxon and Mann-Whitney U test. Parametric data is shown as average ± standard deviation (SD). Association among VAS score, gender, and body mass index was carried out using the Spearman’s correlation test. A *p*-value < 0.05 was accepted as statistically significant.

## Results

Twenty-nine patients, 10 males (34%) and 19 females (66%) were included in the study. The average age, weight, height and body mass index (BMI) of all patients was 53.45 ± 9.6 years, 82.59 ± 12.12 kg, 166.21 ± 8.8 cm and 29.55 ± 4.21, respectively. Body mass index was over 25 in 23 of 29 patients (79.3%). The onset of pain was greater than 1 year in 23 (79.3%) patients and less than 1 year in 6 patients (20.7%) (Table 1). Seventeen patients (58.6%) reported pain after falling backwards, and 4 (13.8%) experienced pain following a traffic accident. In 2 patients (6.9%) the pain was due to cancer and in 6 patients (20.7%) it was idiopathic (Table 1). X-ray evaluations were normal in 10 patients (34.5%), while an anterior coccygeal angulation was found in 19 patients (65.5%) (Table 1).

**Table 1:** Examination findings from 29 patients with coccydynia

*Weight characteristics*	n (%)
BMI < 25	6 (20.7)
BMI 25–29.9	8 (27.6)
BMI ≥ 30	15 (51.7)
*Time of pain onset*	
< 1 year	6 (20.7)
> 1 year	23 (79.3)
*Causes of pain*	
Falling	17 (58.6)
Traffic accident	4 (13.8)
Neoplasm	2 (6.9)
Idiopathic	6 (20.7)
*X-ray findings*	
Normal	10 (34.5)
Anterior angulation	19 (65.5)

Twenty patients (68.9%) underwent DB only, while 9 (31.1%) underwent DB+ pulse RFT. In all patients, a DB with a total of 10 ml of 0.25% bupivacaine+ 40 mg methylprednisolone was administered during the first procedure. The block was performed using either a transsacrococcygeal or intercoccygeal approach.

The average VAS score before undergoing the impar ganglion block was 7.4 ± 1 and decreased to 5.2 ± 1.5 following the procedure. At the 3-month evaluation period, the average VAS score was 4.7 ± 2.1, and at the 3–6 month and 6 month-1 year evaluations the scores were 4.3 ± 2.4 and 4.2 ± 2.6, respectively. VAS scores were found to be significantly lower in patients at the 3-month, 3–6 months and 6 months-1 year follow-up evaluations (*p* < 0.05) (Table 2). VAS scores before the block, after the block and in the first 3-month follow-up period were similar in both the DB block and DB+ pulse RFT block groups. However, patients who received RFT had significantly lower VAS scores at the 3–6 months and 6 months-1 year follow-up periods (Table 3). In these periods, VAS score was lower than 4 in 9 patients who received DB block and in 8 patients who received DB + pulse RFT block. Percentage of improvement in pain score after treatment were 40% in DB block and 72% DB + RFT block. The patients were allowed to take gabapentin, pregabalin or a single tablet of tramadol and paracetamol (37.5 mg + 325 mg) combination when required. In 4 patients, the VAS scores remained ≥7 throughout all of the follow-up measurements (3 patients in the DB group and 1 patient in the DB + pulse RFT group). These patients were referred to surgery for coccyx excision.

**Table 2:** Average VAS scores of impar ganglion block patients

Observation time	VAS (Mean ± SD)	*P*-value
Before the block	7.4 ± 1	
After the block	5.2 ± 1.5	< 0.001
< 3 months	4.7 ± 2.1	< 0.001
3–6 months	4.3 ± 2.4	< 0.001
6 months-1 year	4.2 ± 2.6	< 0.001

*VAS* Visual Analog Scale; Values compared with the average value before the block *p* < 0.01

**Table 3:** Comparison of average VAS scores in diagnostic and radiofrequency impar ganglion block groups

Observation time	DB (*n* = 20)	DB + RFT (*n* = 9)	*P*-value
Before the block	7.4 ± 0,9	7.5 ± 1.1	0.729
After the block	5.5 ± 1.5	4.5 ± 1.4	0.127
< 3 months	5.2 ± 2.3	3.7 ± 1.2	0.085
3–6 months	5 ± 2.5	3 ± 1.8	0.044
6 months-1 year	5 ± 2.4	2.4 ± 2.1	0.010

*DB* Diagnostic block, *RFT* Radiofrequency thermocoagulation

There was no correlation between VAS scores and gender or BMI.

## Discussion

A 1-year follow-up of coccydynia patients demonstrated that the impar ganglion block procedure effectively controlled pain and that a DB + pulse RFT further prolonged analgesic efficacy. Coccydynia is a symptom with different causal factors including twisting of the sacrococcygeal joint or intercoccygeal segment, fracture, infection, tumor or degenerative changes [2]. These factors can lead to localized pain at the coccygeal site, which increases while in a seated position. Pain is the diagnostic symptom, especially in the coccyx and sacrococcygeal areas, as demonstrated during physical examination and through radiologic screening. Conservative treatments include oral analgesic drugs, use of a sitting cushion, physiotherapy, massage, psychotherapy and manipulation [6]. An impar ganglion block is an option when conservative treatment is insufficient; therefore, in this study, we evaluated the efficacy of the impar ganglion block in patients who did not benefit from conservative treatment.

The impar ganglion is the terminal division of the sympathetic chain located in the midline and, in contrast to other divisions, is the only solitary autonomic division. The definitive anatomic location of the impar ganglion remains uncertain but is considered to be anterior to the sacrococcygeal joint, coccyx or distal end of the coccyx [7]. Different approaches to accessing the ganglion using screening techniques such as fluoroscopy, ultrasonography and computerized tomography to view the tip of the needle during the procedure have been described in the literature [8–10]. The sacrococcygeal joint is closed in 51% of patients, which makes access from the intercoccygeal area easier for the physician [11]. The blocks in this study were performed using transsacrococcygeal and intersacrococcygeal approaches with the assistance of fluoroscopy.

Coccydynia occurs more frequently in females. While it can occur at any age, the incidence increases in people over forty [12]. Consistent with the literature, 66% of our patients were female and the average age was 53.45 ± 9.6. A high BMI is also a known risk factor for coccydynia, and, in our study, the average patient had a BMI of 29.55 ± 4.21, which is close to the obesity cut-off value [13]. We observed a tendency of patients to be overweight and obese, but there was no association between the BMI and patient’s VAS scores.

Gündüz et al. noted that trauma, especially after a backwards fall, was the cause of coccydynia in 50% of patients [14]. Similar to this finding, trauma was the leading cause of coccydynia in our study: 58.6% experienced a fall and for 13.8%, a traffic accident was the origin. X-rays and magnetic resonance imaging are useful tools for diagnosing sarcococcygeal hypermobility, hypomobility, fracture and luxation, especially in cases of trauma [15]. In our patients, only X-ray images could be evaluated and were found to be normal in 10 patients (34.5%), while coccygeal angulation at the distal end was observed in the remaining 19 patients (65.5%).

Pain relief can be achieved with conservative medical treatment in 90% of patients with coccydynia [16, 17]. Patients referred to our policlinic were treated with multiple analgesics including NSAIDs, paracetamol and seating cushions. An impar ganglion block was performed if the VAS score remained ≥4 despite conservative treatment. Under fluoroscopy, 20 patients (68.9%) received only DB, and 9 patients (31.1%) received DB + pulse RFT at 42 °C for 2 min at the impar ganglion area. Gonnade et al. reported a significant decrease in pain in 31 patients following impar ganglion blocks in a 1-year follow-up study [18]. We observed a similar significant reduction in pain scores in our patients. The average VAS score was 7.4 ± 1.6 before the block, which then significantly decreased in the first 3 months, 3–6 months and 6 months-1 year evaluations post-procedure.

Radiofrequency treatment is a percutaneous and minimally invasive procedure that can be used in coccydynia patients who do not benefit from appropriate medical treatment and physiotherapy. It is more selective compared to phenol and alcohol neurolysis and leads to fewer complications. Reig et al. reported impar ganglion blocks with RFT as useful for non-malignant perineal pain [8]. Similarly, Kırcelli et al. observed a significant decrease in pain scores, without complications, after impar ganglion block with RFT in patients with chronic coccydynia, except in 2 patients for whom the decrease in pain scores after RFT was no more than 50% [19]. In our study, 4 patients (3 in the DB and 1 in the DB+ pulse RFT group) did not benefit from treatment and were referred to coccyx-excision surgery.

Serious complications such as rectal perforation, hemorrhage and infection, along with difficulties in implementing the technique, restrict the popularity of this highly effective block [6]. In all of our patients, the blocks were performed after both the needle tip and the spread of the radiocontrast substance was confirmed at the relevant area under fluoroscopy. No complications occurred during or after the procedure.

Surgical excision of the coccyx for the treatment of coccydynia is the option of last resort and is performed only if all other treatments have failed. High complication rates and failure to relieve pain have been reported with this procedure, but its overall success rate is between 51 and 90% [15, 20]. In 4 of our patients who underwent surgical coccyx excision due to insufficient pain relief with conservative and interventional treatments, their VAS scores decreased to ≤2 following surgery in 2 patients but remained between 5 and 6 in the other 2.

Limitations to our study include its relatively small sample size and the retrospective nature of the review. The impar ganglion block provides effective pain relief without complications in patients with coccydynia. Diagnostic blocks combined with pulse RFT achieved prolonged analgesic efficacy. However, prospective randomized studies involving larger sample sizes are needed to reach more conclusive results regarding the effectiveness of these procedures.

## Conclusions

İn conclusion, impar ganglion block is an effective procedure with low complication rate for pain relief in coccydynia. The analgesic effect can be prolonged by combining the DB with pulse RFT.

## Data Availability

The datasets used and/or analysed during the current study are available from the corresponding author on reasonable request.

## Abbreviations

NSAIDs: Non-steroidal, anti-inflammatory drugs
RFT: Radiofrequency thermocoagulation
VAS: Visual analog scale
DB: Diagnostic impar block
BMI: Body mass index
